# Evaluation of the acceptability of Peer Physical Examination (PPE) in medical and osteopathic students: a cross sectional survey

**DOI:** 10.1186/1472-6920-13-111

**Published:** 2013-08-22

**Authors:** Fabrizio Consorti, Rosaria Mancuso, Annalisa Piccolo, Giacomo Consorti, Joseph Zurlo

**Affiliations:** 1 Faculty of Medicine and Dentistry, Department of Surgical Sciences, University “Sapienza” of Rome, Viale del Policlinico, 00161, Rome, Italy; 2 Centre pour l’Etude, la Recherche et la Diffusion Ostéopathiques of Rome, Rome, Italy

**Keywords:** Peer physical examination, Embodiment, Medical student, Osteopathic student, Cross sectional survey

## Abstract

**Background:**

Peer physical examination (PPE) is a method of training in medical and osteopathic curricula. The aim of this study was to compare the acceptability of PPE in two classes of medical and osteopathic students after their first experience, to obtain comparative information useful for an understanding of the different professional approaches. The leading hypothesis was that osteopathic students enter the curriculum with a more positive attitude to bodily contact.

As a secondary aim, this study validated the new version of a questionnaire to assess the acceptability of PPE.

**Methods:**

A new version of a previously validated questionnaire and an instrument from the literature (the *Examining Fellow Student* [EFS] questionnaire) were used for a cross-sectional survey in a class of 129 3rd year medical students and in two parallel classes of 1st year osteopathic students (total of 112 students).

**Results:**

The mean score of the new questionnaire was significantly higher for the osteopathic students than for the medical students (53.4 ± 6.3 vs. 43.4 ± 8.9; p < 0.01). The only independent variables that were significantly predictive of the score in a linear regression analysis were gender and the condition of medical or osteopathic student. The EFS mean score also showed a significant difference between the osteopathic and medical students (30.76 ± 2.9 vs. 27.85 ± 4.3; p < 0.01).

Factor analysis of the new questionnaire identified three factors (appropriateness and usefulness, sexual implications and passive role) accounting for 62.8% of the variance. Criterion validity was assessed by correlation with the EFS (Pearson’s r coefficient = 0.61). Reliability was expressed in terms of Cronbach’s alpha coefficient, which equals 0.86.

**Conclusions:**

These quantitative results are consistent with previous qualitative research on the process of embodiment both in medicine and osteopathy. The new questionnaire proved to be valid and reliable. The objective assessment of the acceptability of PPE is a way to determine differences in students’ attitudes towards contact with the body and can be used for counselling students regarding career choice. This study can also highlight differences between students from different professions and serve as a basis for reflection for improved mutual interprofessional understanding and future interprofessional education.

## Background

In many countries, osteopathy is a recognised form of healthcare that relies on manual contact for diagnosis and treatment [1]. In Italy, schools of osteopathy have existed for 30 years, and osteopathic practice is spreading and constitutes one of several forms of complementary and alternative medicine (CAM). Nevertheless, osteopathy in Italy is in the process of being acknowledged as an officially recognised healthcare profession; to overcome a lack of a professional registration body established by the government, the Register of Italian Osteopaths [2] was constituted to act as a self-regulatory body for promoting professional ethics, education and scientific development.

The principles and objectives of osteopathy are not currently taught in Italian medical curricula; although both the medical and osteopathic professions acknowledge the value of interprofessional collaboration [2,3], there are few examples of integration between Italian osteopathic and medical schools. The Faculty of Medicine and Dentistry (FMD) of Sapienza University of Rome and a school of osteopathy, the Centre pour l’Etude, la Recherche et la Diffusion Ostéopathiques (CERDO) of Rome, began a scientific and educational collaboration in order to learn about each other, develop a base of mutual acknowledgement and overcome possible prejudices.

Peer physical examination (PPE) is the learning activity in which students act as models for each other in learning skills in physical examination and simple non-invasive procedures [4]. This technique has also been used for teaching and learning anatomy [5]. PPE is a method to improve students’ skills while avoiding the use of actors or actual patients as models or subjects in physical examinations. The FMD introduced PPE some years ago in its course titled *Introduction to Clinical Medicine* in the 3rd year of a six-year-long program of study. PPE is also a basic method used in osteopathic schools to train students in osteopathic manipulative treatment (OMT) from the beginning of the curriculum.

PPE is a widespread educational practice, and most medical students are comfortable with PPE [6-9]; however, published papers have periodically raised concerns regarding its acceptability [9-11]. Evidence has been collected on the influence of gender on the acceptability of PPE [9,12-16]. Usually women are more reluctant to engage in PPE, especially with students of the opposite gender. Religious beliefs [6,15] and geographic area of origin [15,17] are additional determinants of the level of acceptability of PPE. PPE is used in the education of different professions, and recent studies focused on the issue of acceptability of PPE from students in medical [18], nursing [19] and physiotherapy schools [20]; however, recent reviews of the latest published studies did not result in data providing interprofessional comparisons [21].

The rationale of our study relies on previous empirical research [22,23] about body work in CAM and the disembodiment process in medical examination. The expression ‘*body work*’ refers to the work of healthcare providers that involves a distinctive and often intimate relation to the bodies of consumers, clients or patients [24]. In his ethnographic research in a school of osteopathy, Gale [22] described the concept of ‘*body talk*’ as the way in which the embodied patient is able to communicate with the practitioner, not only through verbal interaction but also through a ‘*dialogue with the tissues*’ during diagnostic palpation and through physical appearance during direct observation. Gale highlighted ‘*the centrality of embodied interaction at the investigative stage of the osteopathic healing process*’.

In a set of case studies, Young [23] examined the phenomenology of the body during a medical examination, arguing that the body is ‘*reframed to exclude some of its symbolic properties*, *especially sexual ones*’. Moreover, the body is transformed into an object of scrutiny in the context of the social contract upon which medical practice relies. The dual attention to the body as incarnate and disincarnate is handled by a delicate ‘*etiquette of touch*’. Gale’s and Young’s qualitative findings suggest a difference in the attitude to bodily contact between medical doctors and osteopaths and led us to hypothesise that students who choose the profession of osteopathy may enter the curriculum with a more positive attitude towards bodily contact.

The objective of this study, then, was to measure the acceptability to PPE among two groups of medical and osteopathic students after their first experience with the technique; the data were obtained to develop comparative information to understand the different professional approaches. The study also aimed to revalidate a revised version of a previously designed questionnaire as an objective instrument of assessment of the acceptability of PPE in students. Both the FMD and CERDO faculties were interested in developing an objective instrument to quickly measure the acceptability of PPE and address some of the components of a relational and cognitive nature. A first version of the questionnaire was developed and validated in a previous study [25]. Because some items were rephrased during the joint process of designing the present study between the FMD and CERDO, we evaluated whether the psychometric features were maintained, particularly across two groups of students in different professions. This is the first large study of the acceptability of PPE among healthcare students in Italy and among the greater Italian-speaking or Latinate areas of Europe.

## Methods

The FMD runs a discipline-based medical curriculum that spans 6 years. After the first two years of basic preclinical science, students encounter clinical subjects during their third year in the course *Introduction to Clinical Medicine*, in which PPE is used to develop their skills in physical examinations. CERDO runs a 6-year long curriculum in osteopathy in conformance with ROI standards. The school offers a full-time curriculum for lay students and a part-time curriculum for medical doctors and physiotherapists. PPE is introduced during the first year as the preferred method for teaching diagnostic palpation and OMT.

We performed a cross sectional survey of a class of 129 3rd year medical students and two parallel classes of 1st year osteopathic students (112 total) enrolled in either the full or part-time curriculum. During the survey, we administered our new questionnaire, and we simultaneously administered the *Examining Fellow Student* (EFS) questionnaire [8] as a point of reference for evaluating criterion validity. The survey was administered in class as a paper-and-pencil task in the academic year 2011-12, at the end of the first semester, during which both groups experienced PPE for the first time. At the beginning of the semester, students were informed that PPE would be used in training and—although the activity is mandatory—they were encouraged to notify the teacher in charge of coordinating the practical activities if they had a strong objection to performing PPE. Medical students performed PPE to develop their skills in physical examinations of the head and neck, chest and heart, and abdomen and legs. Osteopathic students performed PPE to learn a basic set of OMT techniques, involving contact with the same body regions as medical students though using different modalities than those used in a medical physical examination. No additional selection criteria were applied beyond status as a student in one of the selected classes, and there was no sampling because all students in the classes were surveyed.

The study received ethical approval from both schools. Data were collected in an anonymous format and with the oral consent of the students.

Comparisons of the mean scores for stratified subgroups among the two groups were performed using the Kruskal-Wallis test because the distribution of the scores was likely to be non-normal, with an acceptable alpha error of <0.05. Correlation of personal and cultural data with the scores on the questionnaires as a dependent outcome variable was analysed using multivariate linear regression. All statistical calculations were performed using Statistica® software.

## Results

The demographics and relevant cultural data of the surveyed students are presented in Table 1.

**Table 1:** Demographics and social-cultural characteristics of the sample

	**Age**	**Gender**		**Religious belief**		**Geographic area**		
		**Males**	**Females**	**Yes**	**No**	**North**	**Centre**	**South**
**Medical Students (N = 129)**	22.1 ± 3.4	42 (32.6)	87 (67.4)	75 (58.2)	54 (41.8)	1(0.8)	84 (65.1)	44 (34.1)
**Full time osteopathic students (N = 30)**	22.6 ± 6.7	21 (70)	9 (30)	20 (66.6)	10 (33.4)	0 (0)	23 (76.7)	7 (23.3)
**Part time osteopathic students (N = 82)**	28.7 ± 6.7	48 (58.5)	34 (41.5)	54 (65.9)	28 (34.1)	3 (3.7)	58 (70.7)	21 (25.6)
**Total (N = 241)**	24.4 ± 6.0	111 (46.1)	130 (53.9)	149 (61.8)	92 (38.2)	4 (1.6)	165 (68.5)	72 (29.9)

Results are expressed as mean ± SD or number (percentage frequency).

The response rate was 100% of the students attending class on the day of the survey, which were 129 out of the 140 enrolled 3rd year medical students (92.14%) and 112 of the 115 enrolled 1st year osteopathic students (97.4%).

### Validation of the instrument

To measure the acceptability of PPE, we used both the EFS questionnaire and a new questionnaire we developed to gain a deeper understanding of the elements composing the overall construct of acceptability of PPE. The EFS questionnaire explored the overall acceptability of performing and undergoing PPE on various body regions, without any other consideration of possible different dimensions of the construct. Our questionnaire was designed to measure two different although related elements on a five-point Likert scale (0 = completely disagree, 4 = completely agree), as follows:

● the acceptability of the practice of PPE, explored in different contexts and potentially problematic situations (active or passive role, exposure of the body, fear of sexual interest, relationship with partners of the same or opposite gender and with the tutor (questionnaire items 1 to 11)

● the students’ opinion of the educational value of the PPE (items 12 to 16)

The scores on the items were assigned equivalent points on a 0 to 4 scale, except for items 3 through 7 and item 12, which were assigned points in a reverse way. The maximum possible score was 64 points.

The questionnaire also contained a few other items that asked students their opinions regarding organisational topics (formation of working groups, written protocol of conduct) and about their personal and cultural characteristics.

In this sample of students, we assessed construct validity by principal components factor analysis and criterion validity by comparison with the score on the EFS questionnaire. Table 2 lists the items and shows the result of the factor analysis, which identified three factors with an eigenvalue of >1, which were interpreted as “appropriateness and usefulness”, “sexual implications” and “passive role”. These three factors accounted for 62.8% of the variance.

**Table 2:** Factor analysis of the questionnair

**Factor Loadings (Varimax normalized) Extraction: Principal components (Marked loadings are >0.45)**			
**ITEMS**	**Factor1**	**Factor 2**	**Factor 3**
**1.** In general, I feel comfortable when performing PPE on a colleague of mine	**0.64**	−0.10	**0.52**
**2.** In general, I feel comfortable when a colleague performs PPE on me	**0.57**	−0.06	**0.63**
**3.** I feel embarrassed if I am undressed for PPE in front of my group of colleagues	0.13	0.11	**0.82**
**4.** I feel embarrassed if I am undressed for PPE in front of my teacher or tutor	0.26	0.12	**0.82**
**5.** I am concerned of being a possible object of sexual interest during PPE	−0.08	**0.60**	0.45
**6.** I am concerned of experiencing possible sexual interest for my colleagues during PPE	−0.02	**0.79**	−0.05
**7.** I am concerned of experiencing possible sexual interest for my teacher or tutor during PPE	0.22	**0.73**	−0.07
**8.** I feel comfortable when performing PPE on a colleague of my same sex	**0.66**	0.06	0.16
**9.** I feel comfortable when performing PPE on a colleague of the opposite sex than mine	**0.68**	−0.04	0.39
**10.** I feel comfortable when PPE is performed on me by a colleague of my same sex	**0.68**	0.01	0.34
**11.** I feel comfortable when PPE is performed on me by a colleague of the opposite sex than mine	**0.59**	−0.06	**0.57**
**12.** It is inappropriate to perform PPE on persons that will be my future colleagues	0.17	**0.75**	0.12
**13.** To perform PPE is an appropriate practice for the education of a medical doctor (osteopath)	**0.75**	0.22	−0.08
**14.** To undergo PPE is an appropriate practice for the education of a medical doctor (osteopath)	**0.78**	0.11	0.17
**15.** In performing PPE I get useful feed back from my colleagues about my skill	**0.74**	0.18	0.09
**16.** It is a sign of professionalism as a student to accept to perform and undergo PPE	**0.67**	0.09	0.17
**Expl.Var**	0.30	0.14	0.18

Factor Loadings (Varimax normalized) Extraction: Principal components (Marked loadings are >0.45).

The Pearson’s r coefficient between the scores on our questionnaire and the scores on the EFS was 0.61; this finding indicates a good correlation. Reliability was assessed as internal consistency, according to the classical item analysis with Cronbach’s alpha coefficient. This coefficient of reliability showed an acceptable value of 0.86.

The mean and standard deviation of the scores from our new questionnaire were, respectively, 48.09 and 9.29 (min: 22; max: 60; median: 50). The Shapiro-Wilk test for normality showed refusal of the hypothesis of normal distribution of values (p < 0.001).

### Results from the new questionnaire

The mean score on the new questionnaire was 43.4 ± 8.9 for medical students vs. 53.4 ± 6.3 for osteopathic students (p < 0.01). This difference was significant when considering either the full-time (51.07 ± 5.6) or the part-time (54.28 ± 6.21) students of osteopathy. The difference between the two groups of osteopathic students was significant as well (p < 0.05). Overall, PPE was viewed as acceptable by a large majority of the surveyed students: only 3% of the students scored 0 or 1 on the first questionnaire item (taking the active role in PPE, all were medical students) and 12% did the same on the second item (passive role, including only 1 osteopathic student). Nevertheless, when the students were asked if PPE was an appropriate practice (items 13 and 14), the numbers of subjects who gave a 0 or 1 score decreased to 1 student (active role) and 4% (passive role).

Overall, women showed a lower mean score on our questionnaire than men (f: 45.5 ± 9.3 vs. m: 51.1 ± 8.2; p < 0.01). This difference was present among the medical students (f: 42.05 ± 8.5 vs. m: 46.3 ± 9.3; p < 0.02) but was not observed among the osteopathic students (f: 52.6 ± 6.7 vs. m: 53.9 ± 6.0; n.s.). Table 3 summarises the results in greater detail.

**Table 3:** Differences in the mean score of the new questionnaire for type of school, gender, religious belief and geographical area

	**Score**	**Gender**	**p**	**Religious belief**	**p**	**Geographic area **^**(a)**^	**p**
**Medical st.**	43.40 ± 8.9 ^1^	m: 46.35 ± 9.3	<0.05	y: 42.84 ± 9.5	n.s.	centre: 43.80 ± 9.6	n.s.
		f : 42.05 ± 8.5		n: 44.09 ± 8.1		south: 43.04 ± 7.7	
**Full time osteopathic st.**	51.06 ± 5.6^2^	m: 52.14 ± 4.7	n.s.	y: 50.65 ± 4.7	n.s.	centre: 51.30 ± 5.9	n.s.
		f : 48.55 ± 7.1		n: 51.90 ± 7.3		south: 52.00 ± 3.1	
**Part time osteopathic st.**	54.28 ± 6.3^2^	m: 54.68 ± 6.3	n.s.	y: 53.81 ± 6.8	n.s.	centre: 54.91 ± 5.3	n.s.
		f : 53.70 ± 6.3		n: 55.33 ± 5.3		south: 53.76 ± 6.8	
**All osteopathic st.**	53.42 ± 6.3^1^	m: 53.91 ± 6.0	n.s.	y: 52.95 ± 6.4	n.s.	centre: 53.81 ± 5.7	n.s.
		f : 52.63 ± 6.7		n: 54.40 ± 6.0		south: 53.32 ± 6.0	

Results are expressed as mean ± standard deviation.

Maximun theoretical score = 64.

(a) –four students coming from northern regions were excluded from the analysis due to the low number, 10 missing values.

1: medical students vs all osteopathic students and separately vs full or part time osteopathic students: p < 0.01;

2: full time vs part time osteopathic students: p < 0.05; n.s.: not significant.

The scores showed only a weak correlation with age (Pearson’s r = 0.26) in the group of osteopathic students. No significant difference in scores was observed between the subgroups stratified by declared religious beliefs and for the area of origin in Italy for any of the classes. The only independent variables significantly predictive of the scores on the new questionnaire with linear regression analysis were gender and the role of the medical or osteopathic student. The best predictive model accounted for 34% of the variance (R^2^ = 0.34, see Table 4).

**Table 4:** Linear regression model for the score of the new questionnaire as dependent variable

**Variable**	**Coefficient**	**St. Error**	**p**
**Intercept**	31,24	2,70	
**Sex (f)**	3.06	1,1	**< 0,01**
**School (osteopathy)**	9,60	1,10	**< 0,01**
**Geographic area (centre)**	0,45	0,92	n.s.
**Religious belief (y)**	−1,30	1,07	n.s.

### Results from the EFS questionnaire

The overall mean score and standard deviation from the EFS questionnaire were, respectively, 29.14 and 4.03 (min: 12; max: 32; median: 31). The distribution of scores was strongly skewed, with a peak towards the upper end, as shown by the value of the median, which is close to the maximum score.

The EFS mean score showed a significant difference between medical and osteopathic students (27.85 ± 4.3 vs. 30.76 ± 2.9; p < 0.01), but there was not a significant difference between full and part-time osteopathic students (30.27 ± 2.6 vs. 30.96 ± 3.0).

Women scored lower than did men among medical students, while a significant gender difference was not observed among either part-time or full-time osteopathic students.

Table 5 summarises the results in greater detail.

**Table 5:** Differences in the mean score of the EFS questionnaire for type of school, gender, religious belief and geographical area

	**Score**	**Gender**	**p**	**Religious belief**	**p**	**Geographic area **^**(a)**^	**p**
**Medical st.**	27.85 ± 4.3^1^	m: 29.56 ± 4.5	<0.01	y: 27.81 ± 4.4	n.s.	centre: 27.60 ± 4.6	n.s.
		f : 27.05 ± 4.1		n: 27.83 ± 4.3		south: 28.39 ± 3.7	
**Full time osteopathic st.**	30.27 ± 2.6	m: 30.85 ± 2.0	n.s.	y: 30.75 ± 2.1	n.s.	centre: 29.90 ± 2.8	n.s.
		f : 28.88 ± 3.3		n: 29.30 ±3.2		south: 31.38 ± 1.9	
**Part time osteopathic st.**	30.96 ± 3.0	m: 31.22 ± 2.7	n.s.	y: 30.88 ± 2.9	n.s.	centre: 30.62 ± 3.6	n.s.
		f : 30.58 ± 3.3		n: 31.08 ± 3.3		south: 31.66 ± 1.0	
**All osteopathic st.**	30.76 ± 2.9^1^	m: 31.11 ± 2.52	n.s.	y: 30.84 ± 2.6	n.s.	centre: 30.39 ± 3.4	n.s.
		f : 30.20 ± 3.3		n: 30.57 ± 3.3		south: 31.57 ± 1.2	

Results are expressed as mean ± standard deviation.

Maximum theoretical score = 52.

(a) –four students coming from northern regions were excluded from the analysis due to the low number, 10 missing values.

1: medical students vs all osteopathic students and separately vs full or part time osteopathic students: p < 0.01; full time vs part time osteopathic students: n.s.

n.s.: not significant.

## Discussion

### Differences between medical and osteopathic students

Our study confirmed the hypothesis that osteopathic students show a higher acceptance of PPE than did medical students after their first experience and that this difference is not explained by any of the demographic or sociocultural variables we considered. This difference was significant in the responses to both our questionnaire and the EFS questionnaire.

These results are consistent with the idea that osteopathic students approach their curriculum with a stronger acceptance of bodily contact. A possible explanation for the difference we observed can be based on the different social expectations of the students of these two professional groups when entering their respective training programs. Students do not begin a training program as blank slates but rather holding certain ideas of professional values and roles. Blue et al. [26] showed that the attitudes of matriculating students are positive regarding several of the attributes associated with traditional definitions of professionalism, even if some incongruent beliefs are present. In a study of two cohorts of matriculating students [27], we observed that the idea of professionalism tends to be technically oriented, especially in students coming from families in which one of the parents was a doctor. We have no information concerning the image that students in their first year of osteopathic training have of their future profession, but it seems plausible that the idea of touching their patients is believed to be a special competence of an osteopath, while there are many specialties in contemporary medicine in which bodily contact is reduced or absent.

In a qualitative analysis of students’ comments concerning PPE, in the light of Engeström model of activity theory [28], the authors noted that the students clearly differentiated between the peer examiner-examinee relationship and the doctor-patient relationship. PPE blurred interpersonal boundaries in an unexpected way, producing ambiguities. Apparently, this is less true of osteopathic students, who in ‘*learning to interact with the bodies of their patients*, *develop a new orientation to their own bodies*’ [22] and are more prone to an inter-subjectivity focused on bodily contact.

When considering the distribution of the scores of our questionnaire in greater detail, students ranking in the lowest quintile (under a score of 40) received, as expected, on average, a lower contribution to their total scores from the items associated with the “appropriateness” and “passive role” subscales (48.6% and 31.8% of the maximum score for each subscale) than did students in the second quintile (67.6% and 47.0%), while the difference in the “sexual” subscale was more limited (85.4% vs. 94.4%). The distribution of the scores for this last subscale was skewed towards the highest scores, and the lowest scores identified a small subset of students not always ranking in the lowest two quintiles of the overall score. This can be an important indication and a topic of personal counselling with students who may have had a past negative sexual experience, as reported by Power et al [9].

Female medical students showed a higher level of concern regarding PPE than did men; this finding is similar to previously reported results [13-16]. This gender difference did not reach statistical significance between the two classes of osteopathic students, even if the female students in the full-time osteopathic curriculum (who were younger than their counterparts in the part-time curriculum and as old as the medical students) tended to show a slightly weaker acceptance of PPE than did the men. In her analysis of PPE, and in accordance with feminist theory, Rees et al. [29] concluded that older women tend to be less comfortable with PPE than younger women. This conclusion is apparently in disagreement with our results, but Rees explained that the concern of older women is that they unfavourably compare themselves with younger women. This was not the case with our two subgroups of osteopathic students, which were each rather uniform as to age and thus avoided the possibility of such comparisons. The difference in mean age and the fact that full-time students were already registered professionals (doctors or physiotherapists) can be possible explanations for the differences observed between full and part-time osteopathic students.

We did not observe that cultural factors, such as religious belief and geographic area of origin, were significant factors, in contrast to previously reported results [15]. Those with strong religious beliefs scored slightly lower than those with weaker beliefs, but the difference was not significant. All students with stronger beliefs were Roman Catholic and, despite the strong influence that the Catholic church has had on the history of Italy, Italy is now less dominated by Catholicism that it once was. Italy is quickly becoming a multi-ethnic country [30] and perhaps in the future, with an increase of students of other religions, the situation could change. We expected a lower acceptance of PPE among students from southern Italy because this region is thought to have traditional views of modesty, but our findings refuted this hypothesis.

### The new questionnaire

Our questionnaire was developed in accordance with the methodology proposed by Wilson [31] in four steps: conceptual definition of the construct, description of the set of items, strategy for coding of the responses, and calibration of the instrument. This process included iterations of discussion with experts outside the project team, and a panel of five members of the faculty was involved. When the cooperation between the FMD and CERDO started, the first version of the questionnaire was revised by a panel of faculty members from CERDO, and some of the items were rephrased. This process was meant to ensure content validity, even if a formal assessment including a content validity index was not performed [32].

With regard to construct validity, factor analysis showed three factors accounting for 62% of total variance. These three components had high factor loadings and suggested a robust structure of the construct, consistent with our hypothesis when we designed the instrument, even if slightly different. The two components of general attitude and perceived educational value identified in the conceptual design went together in the first factor, while two other rather independent components emerged, connected with sexual issues and the passive role of exposing one’s own body to physical examination by a peer. The first factor was somehow expected. The latter can be connected to what other researchers [33] denoted as embarrassment (from a student’s interview: *‘I just think I’m embarrassed about my body image*, *my body and people seeing it’*), a construct that is not directly linked to sexuality but rather to cultural notions of body image.

Some items had similar loadings across two factors (items 1, 2, 5 and 11), and this finding may reflect the complexity of the underlying construct. In three out of the four items, the two overlapping factors were the “appropriateness and usefulness” and “passive role”, which share a common meaning that the instrument fails to discriminate.

Although the EFS questionnaire cannot be considered a gold standard, it is currently the most used tool in the literature to measure the acceptability of PPE; thus, we thought it could serve as a good point of reference for criterion validity. The EFS questionnaire and our questionnaire differ in terms of their basic conceptual structure: the EFS is used to evaluate the performance of PPE focusing on various body areas, whereas ours is used to investigate global perceptions and beliefs. EFS contains a free response section, in which students are to write comments. These comments have been qualitatively analysed [34] and the emerging themes were similar to the topics we assessed with our questionnaire. Even if the two questionnaires differ in their approaches (more behavioural in the EFS, more cognitive in our questionnaire), it is noteworthy that the correlation of the scores was high. In fact, compared to the EFS, our questionnaire is more similar to the one used by Power et al. [9], although it is shorter and it does not address sensitive areas of the body (breast, genitals, perineum), which are excluded in our protocol for PPE.

### Limitations

This study has some limitations. The first one is that it is based on a convenience sample, introducing a possible bias and limiting the possibility of fully exploring some of the variables, such as the geographic origin. Only 4 students were from northern Italy, and therefore this subgroup could not be considered large enough for valid analysis. The absence of a sampling strategy also prevented correct calculation of the dimensions of the sample. Hence some conclusions, particularly regarding the absence of effect, must be considered cautiously. More robust conclusions could be derived from a larger, more ethnically diverse, nationwide study.

A second limitation is the design of the cross-sectional study. The construct of the acceptability of PPE may vary over time during the curriculum, and this should be considered in the interpretation of our data, which must considered valid only as an instantaneous measure of attitudes at the end of the semester after the first experience with PPE. Some other studies investigated the longitudinal variations with time in acceptability of PPE [15,19,33], reporting contradictory findings. Rees et al. [15] reported that students’ attitudes towards PPE was generally stable during their first year of training, even if a slight but significant decline was noted in the number of body parts students were willing to examine in peers of the same or opposite gender. On the contrary, an increase was noted in the willingness to have some areas of the body examined by peers. In a qualitative study based on focus groups with students whose scores on the EFS changed after a year of practicing PPE, McLachlan et al. [33] reported that students downplayed the significance of the changes. The authors concluded that changes should be investigated with specifically designed qualitative studies. Finally, in their study of nursing students [19], Wearn et al. found that 3rd year students showed higher levels of comfort with PPE than did 1st year students, even if this finding is not from a longitudinal study and—as we showed in our study—the results of a study on one professional group should be applied to another profession with caution.

A second consequence of our cross-sectional design is that we cannot provide data regarding the reproducibility of (e.g., test-retest reliability) and responsiveness to changes in the questionnaire. These topics are open to discussion, especially in view of the routine use of the instrument in formative assessment, and they need to be addressed with a specific longitudinal study.

A third possible limitation could be the differences in years of the curriculum. We compared 1st year osteopathic students to 3rd year medical students; this can imply a difference in age and a possible impact of previous experiences. A first observation is that medical students were as old as full-time osteopathic students (22.1 ± 3.4 vs. 22.6 ± 6.7), while the part-time osteopathic students were older (28.7 ± 6.7) because they were already graduated healthcare professionals. The difference in scores between the two subgroups of osteopathic students could then be related to a difference in age and professional experience, as already discussed and in agreement with the above-mentioned results for nursing students [19]. Nevertheless, full-time osteopathic students scored higher than medical students with the same mean age. We chose the 3rd and 1st years of the curriculum because these are the years in which the practice of PPE begins. In the first two years, medical students at the FMD only attend lecture and laboratory classes; these teaching activities are unlikely to influence their attitudes to PPE. We have no information about possible previous learning or working experiences of full-time osteopathic students, who in the 1st year are as old as 3rd year medical students because the choice of an osteopathic school is usually a second option.

## Conclusions

Overall, PPE was acceptable to medical and osteopathic students, although to different extents. Although 12% of medical students felt embarrassed while undergoing PPE, and only 4% considered it inappropriate. There is still much debate regarding whether PPE should be a mandatory or elective learning activity [4,11]. Our data from our sample of Italian students showed that there are no strong constraints to the use of PPE, but this activity should nevertheless be carefully designed and introduced before being implemented.

The FMD and CERDO started their cooperation with the comparative evaluation of a teaching/learning activity that we thought would be able to yield information regarding the inner nature of the two professional processes of allopathic medicine and osteopathy. The results of this study were also meant to validate an instrument to be used in routine formative assessment.

At the FMD, PPE is performed throughout the 3rd year to train students in physical examination skills, so the scores on the questionnaires at the end of the first semester will be used to identify students with possible issues with PPE after their first experience with the technique. The FMD established a framework for the assessment of professionalism, according to a multimodal longitudinal perspective [35]. Data from the questionnaire regarding students’ experiences with PPE will be integrated into this framework because these data can contribute to developing a portrait of each student’s attitudes and can be used in counselling students regarding their future choice of career.

At CERDO, it is important to identify, as early as possible, students with serious concerns regarding contact with the body because OMT is a core competence in osteopathic practice and PPE is taught and used throughout the 6 years of the curriculum.

Contact with the body, in particular PPE, proved to be a topic of study valuable to this goal of CERDO, and the results of this study – which have been presented to students and discussed during a joint meeting among teachers – may contribute to the reflection by teachers and students on their own practice and to mutual understanding and acknowledgement between the osteopathic and medical professions. The experience of collaboration between the two schools in this project served as a basis for further joint learning and research activities with the aim of developing guidelines and scientific evidence for further collaborative patient-centred practice.

## Competing interests

The authors declare that they have no competing interests.

## Authors’ contributions

FC conceived and coordinated the study, performed the statistical analysis and drafted the manuscript. Questionnaires were administered and the scores were calculated by RM and AP for medical students and by GC and JZ for osteopathic students. All authors participated in the design, data analysis and helped to revise the manuscript. All authors read and approved the final manuscript.

## Pre-publication history

The pre-publication history for this paper can be accessed here:

http://www.biomedcentral.com/1472-6920/13/111/prepub

## References

[B1] American Association of Colleges of Osteopathic Medicine and the American Osteopathic Association Osteopathic medical education in the United States: improving the future of medicine 2005 Washington, DC: A report jointly sponsored by the American Association of Colleges of Osteopathic Medicine and the American Osteopathic Association

[B2] Registro degli Osteopati Italiani [ROI] Code of professional conduct of Italian Osteopath http://www.registro-osteopati-italia.com/wp-content/uploads/2010/12/CODICE-DEONTOLOGICO-2010.pdf

[B3] Federazione Nazionale degli Ordini dei Medici, Chirurghi e degli Odontoiatri [FNOMCEO] Code of professional conduct of Italian Medical Doctors http://www.fnomceo.it/fnomceo/Codice+di+Deontologia+Medica+2006.html?t=&id=3694

[B4] Outram S Nair BR Peer physical examination: time to revisit? Med J Aust 2008 189 5 274 276 18759726 10.5694/j.1326-5377.2009.tb02437.x

[B5] Chinnah TI de Bere SR Collett T Students’ views on the impact of peer physical examination and palpation as a pedagogic tool for teaching and learning living human anatomy Med Teach 2011 33 1 e27 e36 10.3109/0142159X.2011.530313 21182371

[B6] O’Neill PA Larcombe C Duffy K Dornan TL Medical students’ willingness and reactions to learning basic skills through examining fellow students Med Teach 1998 20 433 437 10.1080/01421599880526

[B7] Chang EH Power DV Are medical students comfortable with practicing physical examinations on each other? Acad Med 2000 75 4 384 389 10.1097/00001888-200004000-00020 10893124

[B8] Rees CE Bradley P McLachlan JC Exploring medical students’ attitudes towards peer physical examination Med Teach 2004 26 1 86 88 10.1080/01421590310001642984 14744701

[B9] Power DV Center BA Examining the medical student body: peer physical exams and genital, rectal, or breast exams Teach Learn Med 2005 17 337 343 10.1207/s15328015tlm1704_5 16197320

[B10] Braunack-Mayer AJ Should medical students act as surrogate patients for each other? Med Educ 2001 35 681 686 10.1046/j.1365-2923.2001.00970.x 11437971

[B11] Rizan CT Shapcott L Nicolson AE Mason JD PPE: a UK perspective, ‘All for one, NOT one for all’ Med Teach 2012 34 1 82 22250683 10.3109/0142159X.2012.640725

[B12] Barnette JJ Kreiter CD Schuldt SS Student attitudes towards same-gender versus mixed-gender partnering in practicing physical examination skills Eval Health Prof 2000 23 360 370 10.1177/01632780022034651 11067196

[B13] Rees CE Bradley P Collett T McLachlan JC “Over my dead body?”: the influence of demographics on students’ willingness to participate in peer physical examination Med Teach 2005 27 7 599 605 10.1080/01421590500237671 16332551

[B14] Wearn A Bhoopatkar H Evaluation of consent for peer physical examination: students reflect on their clinical skills learning experience Med Educ 2006 40 10 957 964 10.1111/j.1365-2929.2006.02557.x 16987185

[B15] Rees CE Wearn AM Vnuk AK Sato TJ Medical students’ attitudes towards peer physical examination: findings from an international cross-sectional and longitudinal study Adv Health Sci Educ Theory Pract 2009 14 1 103 121 10.1007/s10459-007-9094-y 18214706

[B16] Chen JY Yip AL Lam CL Patil NG Does medical student willingness to practise peer physical examination translate into action? Med Teach 2011 33 10 e528 e540 10.3109/0142159X.2011.599893 21942489

[B17] Das M Townsend A Hasan MY The views of senior students and young doctors of their training in a skills laboratory Med Educ 1998 32 2 143 149 10.1046/j.1365-2923.1998.00182.x 9743765

[B18] Reid KJ Kgakololo M Sutherland RM Elliott SL Dodds AE First-year medical students’ willingness to participate in peer physical examination Teach Learn Med 2012 24 1 55 62 10.1080/10401334.2012.641489 22250937

[B19] Wearn AM Bhoopatkar H Mathew TK Stewart L xploration of the attitudes of nursing students to peer physical examination and physical examination of patients Nurse Educ Today 2012 S0260-6917(12)00275-4. 10.1016/j.nedt.2012.08.012 10.1016/j.nedt.2012.08.012 22986173

[B20] Delany C Frawley H A process of informed consent for student learning through peer physical examination in pelvic floor physiotherapy practice Physiotherapy 2012 98 1 33 39 10.1016/j.physio.2011.04.347 22265383

[B21] Hendry GJ Barriers to undergraduate peer-physical examination of the lower limb in the health sciences and strategies to improve inclusion: a review Adv Health Sci Educ Theory Pract 2012 10.1007/s10459-012-9418-4 23073624

[B22] Gale NK From body-talk to body-stories: body work in complementary and alternative medicine Sociol Health Iln 2011 33 2 237 251 10.1111/j.1467-9566.2010.01291.x 21029118

[B23] Young K Disembodiment: the phenomenology of the body in medical examinations Semiotica 1989 1/2 43 66

[B24] Wolkowitz C Bodies at work 2006 London: Sage

[B25] Consorti F Mancuso R Milazzo F Notarangelo MG Piccolo A Acceptability among Italian medical students of Peer Physical Examination (PPE): validation of tools of measure and first results of a survey Tutor 2012 12 1 16 25

[B26] Blue AV Crandall S Nowacek G Luecht R Chauvin S Swick H Assessment of matriculating medical students’ knowledge and attitudes towards professionalism Med Teach 2009 31 10 928 932 10.3109/01421590802574565 19877866

[B27] Consorti F Potasso L Toscano E L’idea di professionalità medica degli studenti di Medicina: rilevazione di base per uno studio di coorte [The concept of medical professionalism of medical students: basic assessment for a cohort study] Clin Ter 2012 163 6 e377 386 23306749

[B28] Wearn AM Rees CE Bradley P Vnuk AK Understanding student concerns about peer physical examination using an activity theory framework Med Educ 2008 42 12 1218 1226 10.1111/j.1365-2923.2008.03175.x 19120953

[B29] Rees CE The influence of gender on student willingness to engage in peer physical examination: the practical implications of feminist theory of body image Med Educ 2007 41 8 801 807 10.1111/j.1365-2923.2007.02779.x 17661888

[B30] Italian Institute of Statistics [ISTAT] Italia in cifre 2012 [Italy in figures 2012] 2012: Rome: ISTAT Pub

[B31] Wilson M Constructing measures: an item response modeling approach 2005 Mahwah, New Jersey: Lawrence Erlbaum Associates Pub

[B32] Polit DF Beck CT The content validity index: are you sure you know what’s being reported?Critique and recommendations Res Nurs Health 2006 29 5 489 497 10.1002/nur.20147 16977646

[B33] McLachlan JC White P Donnelly L Patten D Student attitudes to peer physical examination: a qualitative study of changes in expressed willingness to participate Med Teach 2010 32 2 e101 105 10.3109/01421590903202504 20163215

[B34] Rees CE Wearn AM Vnuk AK Bradley PA Don’t want to show fellow students my naughty bits: medical students’ anxieties about peer examination of intimate body regions at six schools across UK, Australasia and Far-East Asia Med Teach 2009 31 10 921 927 10.3109/01421590802578244 19877865

[B35] Consorti F Notarangelo M Potasso L Toscano E Developing professionalism in Italian medical students: an educational frame work Adv Med Educ Practice 2012 3 55 60 10.2147/AMEP.S31228 PMC3650871 23762002

